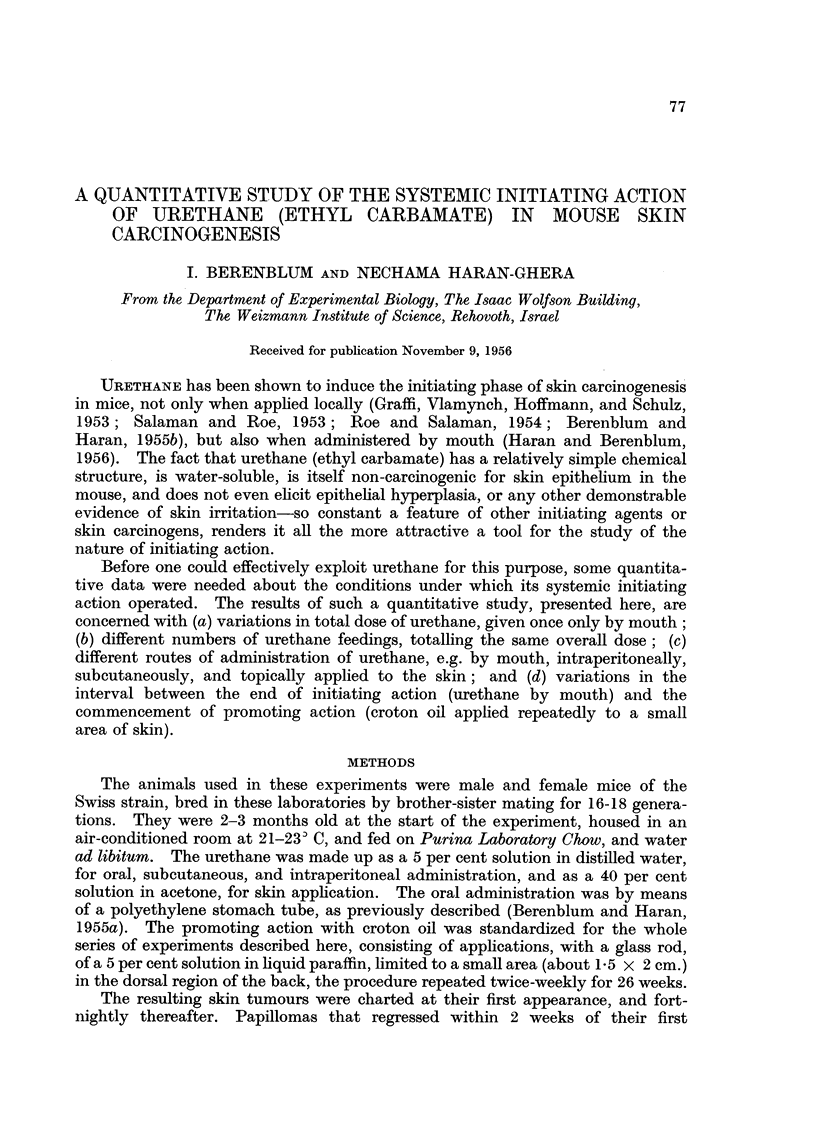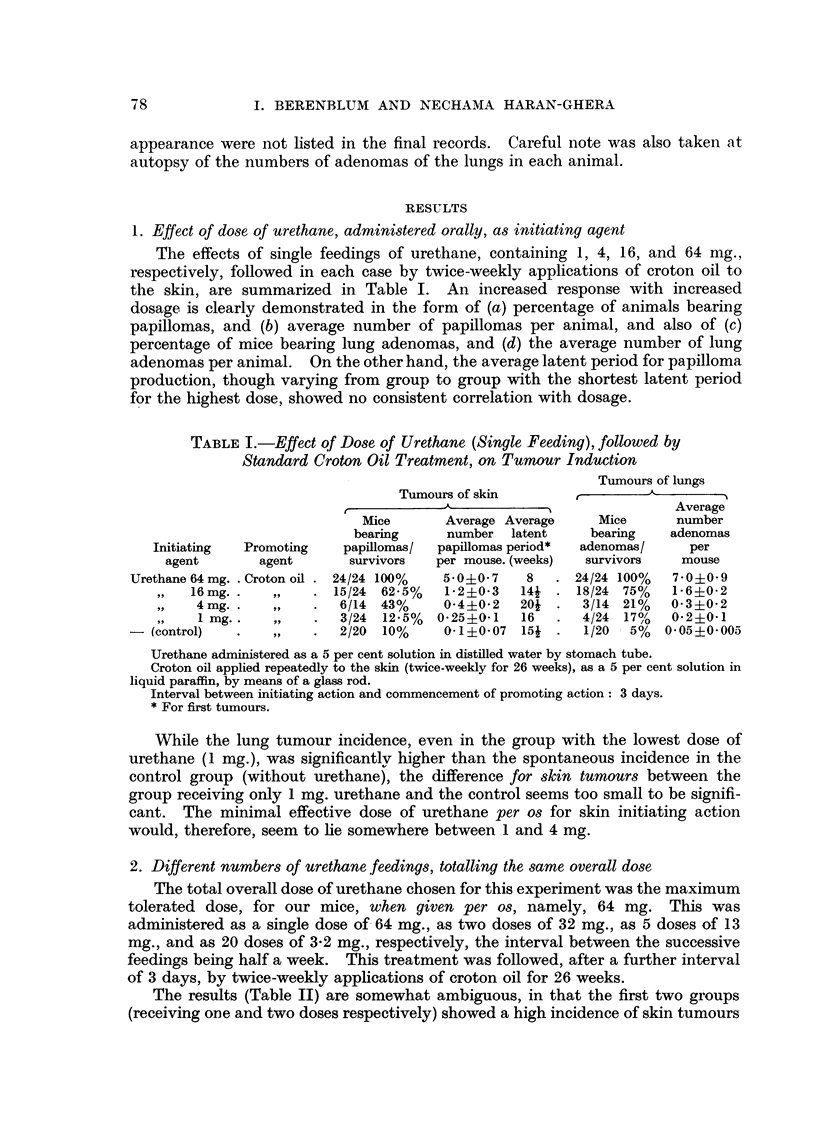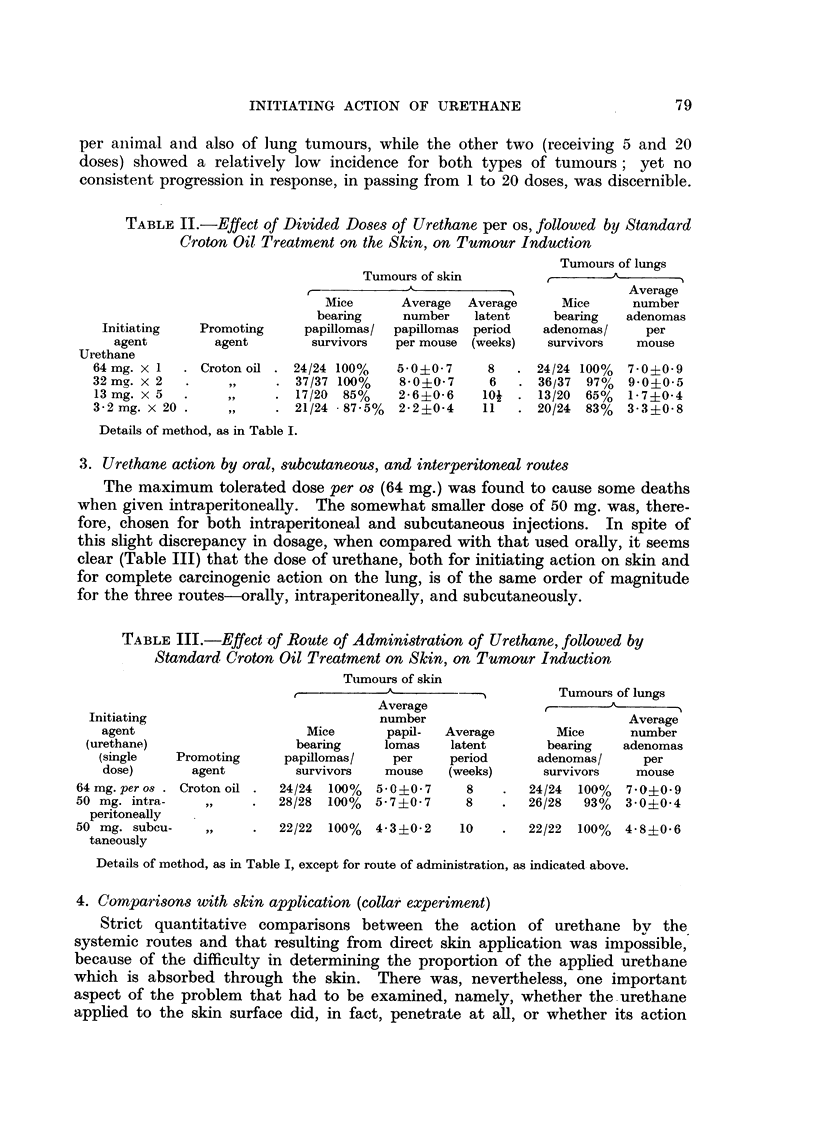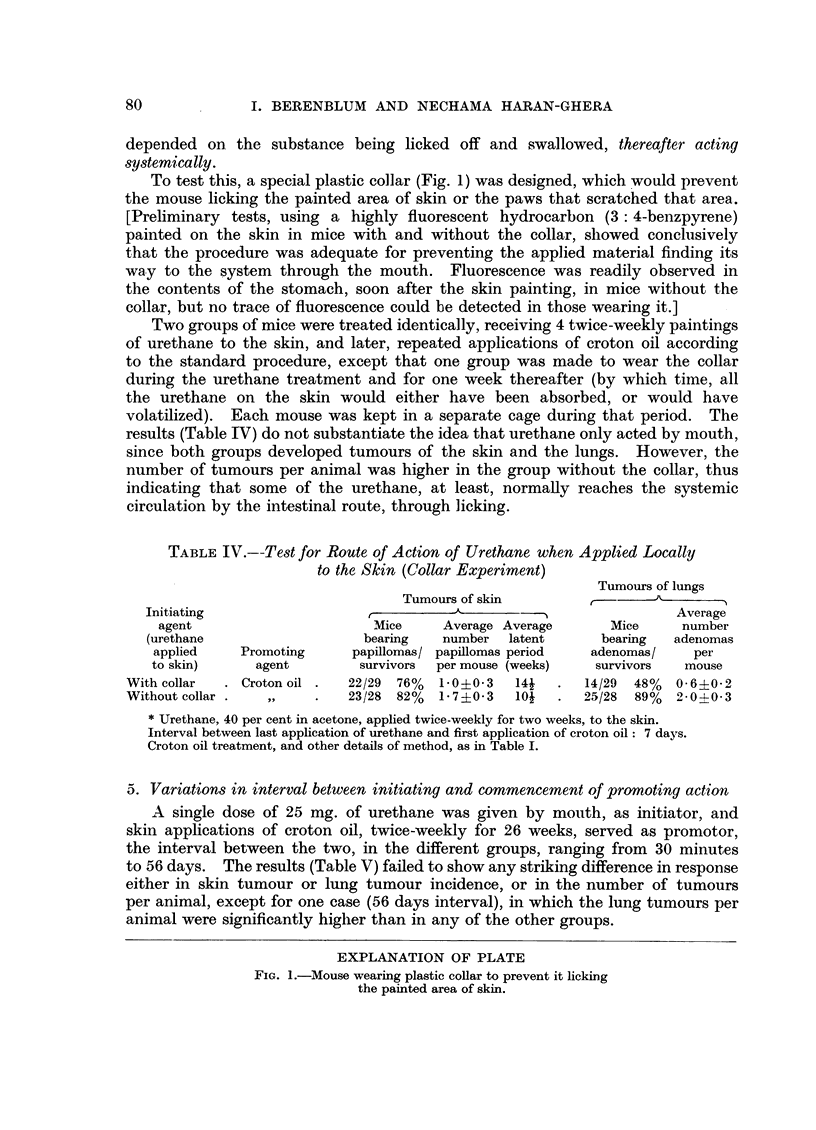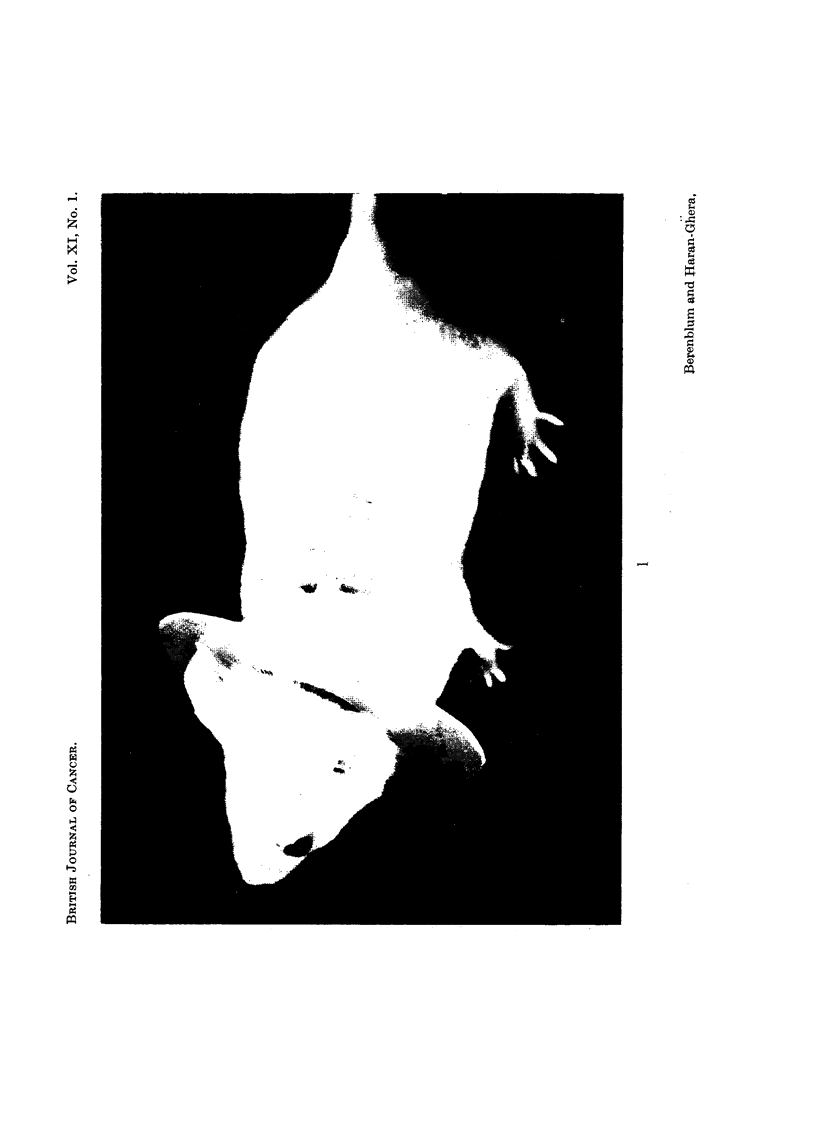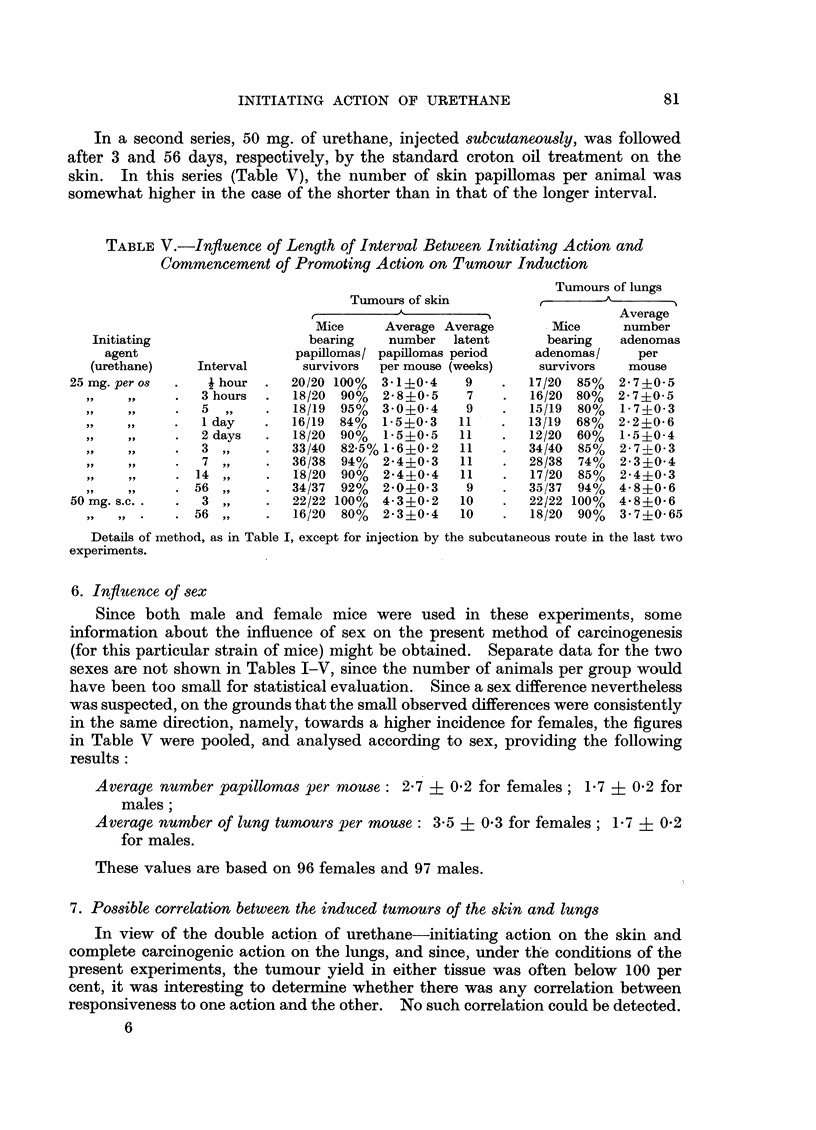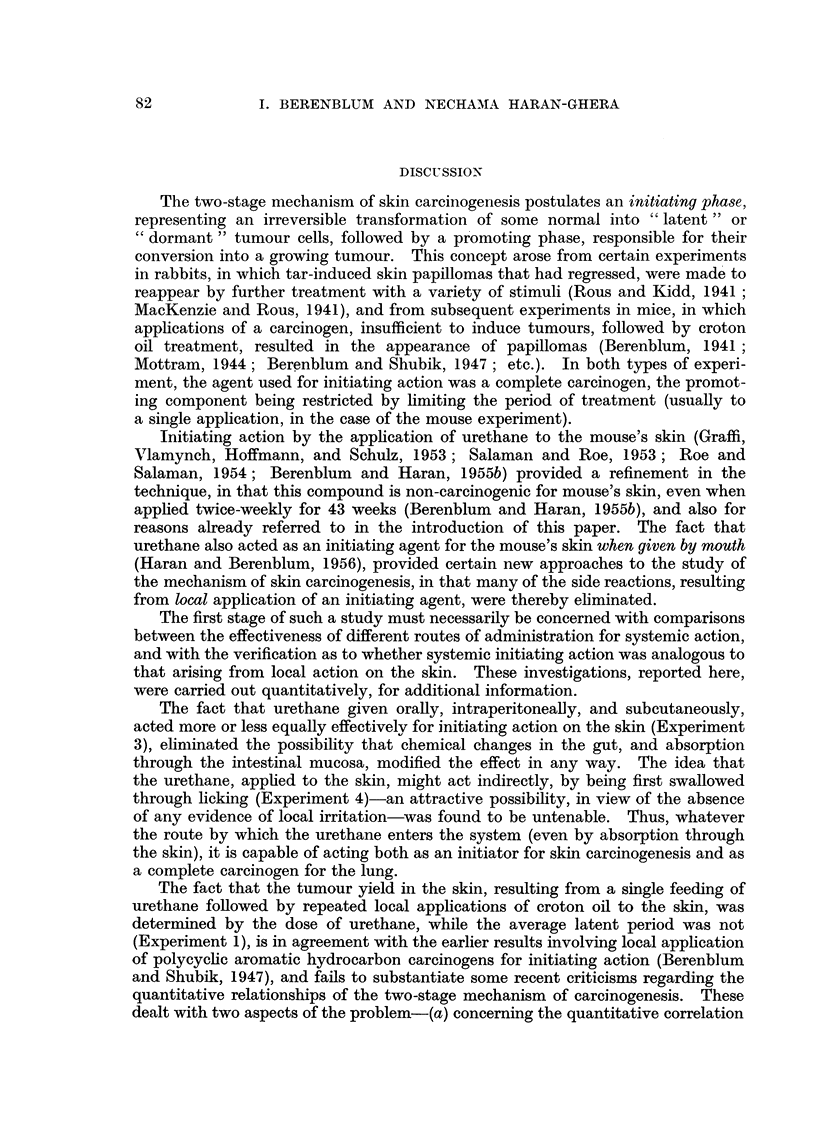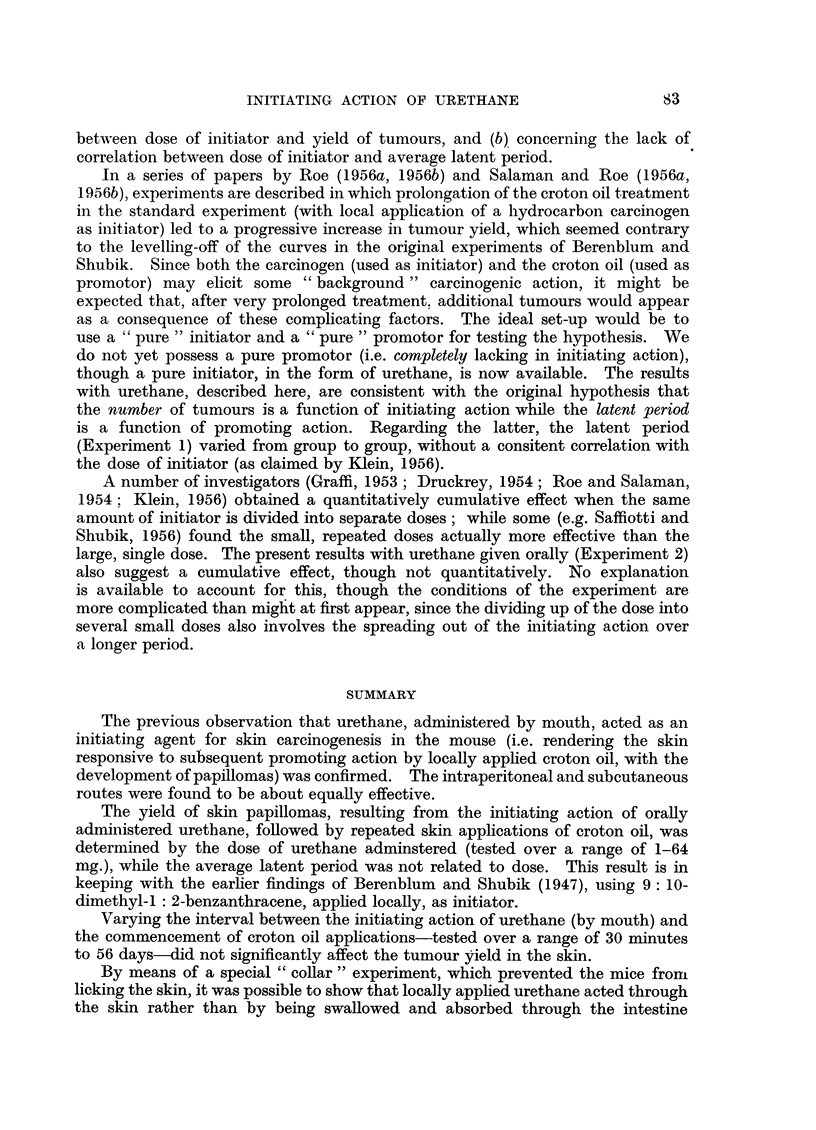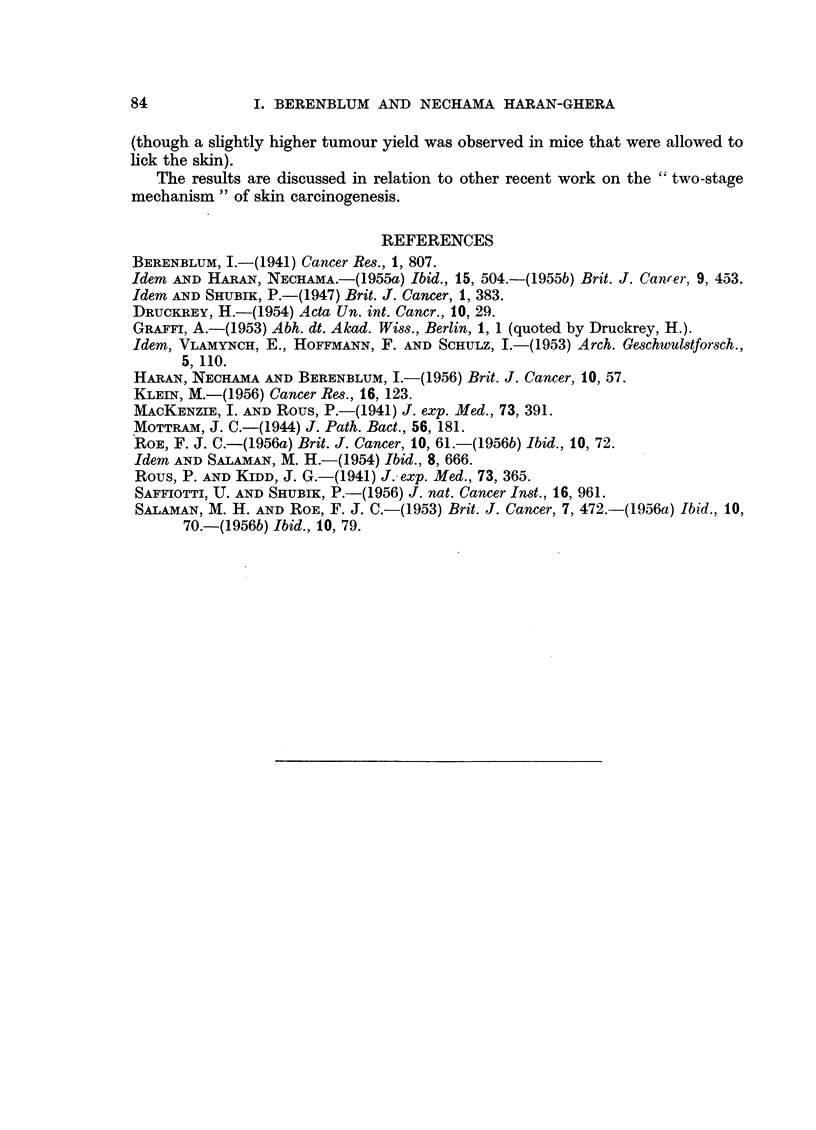# A Quantitative Study of the Systemic Initiating Action of Urethane (Ethyl Carbamate) in Mouse Skin Carcinogenesis

**DOI:** 10.1038/bjc.1957.11

**Published:** 1957-03

**Authors:** I. Berenblum, Nechama Haran-Ghera

## Abstract

**Images:**


### A QUANTITATIVE STUDY OF THE SYSTEMIC INITIATING ACTION OF URETHANE (ETHYL CARBAMATE) IN MOUSE SKIN CARCINOGENESIS

I. BERENBLUM AND NECHAMA HARAN-GHERA

*From the Department of Experimental Biology, The Isaac Wolfson Building, The Weizmann Institute of Science, Rehovoth, Israel*

Received for publication November 9, 1956

URETHANE has been shown to induce the initiating phase of skin carcinogenesis in mice, not only when applied locally (Graffi, Vlamynch, Hoffmann, and Schulz, 1953; Salaman and Roe, 1953; Roe and Salaman, 1954; Berenblum and Haran, 1955*b*), but also when administered by mouth (Haran and Berenblum, 1956). The fact that urethane (ethyl carbamate) has a relatively simple chemical structure, is water-soluble, is itself non-carcinogenic for skin epithelium in the mouse, and does not even elicit epithelial hyperplasia, or any other demonstrable evidence of skin irritation—so constant a feature of other initiating agents or skin carcinogens, renders it all the more attractive a tool for the study of the nature of initiating action.

Before one could effectively exploit urethane for this purpose, some quantitative data were needed about the conditions under which its systemic initiating action operated. The results of such a quantitative study, presented here, are concerned with (*a*) variations in total dose of urethane, given once only by mouth; (*b*) different numbers of urethane feedings, totalling the same overall dose; (*c*) different routes of administration of urethane, e.g. by mouth, intraperitoneally, subcutaneously, and topically applied to the skin; and (*d*) variations in the interval between the end of initiating action (urethane by mouth) and the commencement of promoting action (croton oil applied repeatedly to a small area of skin).

### METHODS

The animals used in these experiments were male and female mice of the Swiss strain, bred in these laboratories by brother-sister mating for 16-18 generations. They were 2–3 months old at the start of the experiment, housed in an air-conditioned room at 21–23° C, and fed on *Purina Laboratory Chow*, and water *ad libitum*. The urethane was made up as a 5 per cent solution in distilled water, for oral, subcutaneous, and intraperitoneal administration, and as a 40 per cent solution in acetone, for skin application. The oral administration was by means of a polyethylene stomach tube, as previously described (Berenblum and Haran, 1955*a*). The promoting action with croton oil was standardized for the whole series of experiments described here, consisting of applications, with a glass rod, of a 5 per cent solution in liquid paraffin, limited to a small area (about 1·5 × 2 cm.) in the dorsal region of the back, the procedure repeated twice-weekly for 26 weeks.

The resulting skin tumours were charted at their first appearance, and fortnightly thereafter. Papillomas that regressed within 2 weeks of their first appearance were not listed in the final records. Careful note was also taken at autopsy of the numbers of adenomas of the lungs in each animal.

### RESULTS

### 1. *Effect of dose of urethane, administered orally, as initiating agent*

The effects of single feedings of urethane, containing 1, 4, 16, and 64 mg., respectively, followed in each case by twice-weekly applications of croton oil to the skin, are summarized in Table I. An increased response with increased dosage is clearly demonstrated in the form of (*a*) percentage of animals bearing papillomas, and (*b*) average number of papillomas per animal, and also of (*c*) percentage of mice bearing lung adenomas, and (*d*) the average number of lung adenomas per animal. On the other hand, the average latent period for papilloma production, though varying from group to group with the shortest latent period for the highest dose, showed no consistent correlation with dosage.

TABLE I.—*Effect of Dose of Urethane (Single Feeding), followed by Standard Croton Oil Treatment, on Tumour Induction*

| | | Tumours of skin | | | | Tumours of lungs | | |
|---|---|---|---|---|---|---|---|---|
| Initiating agent | Promoting agent | Mice bearing papillomas/ survivors | | Average number papillomas per mouse. | Average latent period* (weeks) | Mice bearing adenomas/ survivors | | Average number adenomas per mouse |
| Urethane 64 mg. | Croton oil | 24/24 | 100% | 5·0±0·7 | 8 | 24/24 | 100% | 7·0±0·9 |
| ,, 16 mg. | ,, | 15/24 | 62·5% | 1·2±0·3 | 14½ | 18/24 | 75% | 1·6±0·2 |
| ,, 4 mg. | ,, | 6/14 | 43% | 0·4±0·2 | 20½ | 3/14 | 21% | 0·3±0·2 |
| ,, 1 mg. | ,, | 3/24 | 12·5% | 0·25±0·1 | 16 | 4/24 | 17% | 0·2±0·1 |
| — (control) | ,, | 2/20 | 10% | 0·1±0·07 | 15½ | 1/20 | 5% | 0·05±0·005 |

Urethane administered as a 5 per cent solution in distilled water by stomach tube.

Croton oil applied repeatedly to the skin (twice-weekly for 26 weeks), as a 5 per cent solution in liquid paraffin, by means of a glass rod.

Interval between initiating action and commencement of promoting action : 3 days.

* For first tumours.

While the lung tumour incidence, even in the group with the lowest dose of urethane (1 mg.), was significantly higher than the spontaneous incidence in the control group (without urethane), the difference *for skin tumours* between the group receiving only 1 mg. urethane and the control seems too small to be significant. The minimal effective dose of urethane *per os* for skin initiating action would, therefore, seem to lie somewhere between 1 and 4 mg.

### 2. *Different numbers of urethane feedings, totalling the same overall dose*

The total overall dose of urethane chosen for this experiment was the maximum tolerated dose, for our mice, *when given per os*, namely, 64 mg. This was administered as a single dose of 64 mg., as two doses of 32 mg., as 5 doses of 13 mg., and as 20 doses of 3·2 mg., respectively, the interval between the successive feedings being half a week. This treatment was followed, after a further interval of 3 days, by twice-weekly applications of croton oil for 26 weeks.

The results (Table II) are somewhat ambiguous, in that the first two groups (receiving one and two doses respectively) showed a high incidence of skin tumours per animal and also of lung tumours, while the other two (receiving 5 and 20 doses) showed a relatively low incidence for both types of tumours; yet no consistent progression in response, in passing from 1 to 20 doses, was discernible.

TABLE II.—*Effect of Divided Doses of Urethane* per os, *followed by Standard Croton Oil Treatment on the Skin, on Tumour Induction*

| Initiating agent | Promoting agent | Tumours of skin: Mice bearing papillomas/survivors | | Tumours of skin: Average number papillomas per mouse | Tumours of skin: Average latent period (weeks) | Tumours of lungs: Mice bearing adenomas/survivors | | Tumours of lungs: Average number adenomas per mouse |
|---|---|---|---|---|---|---|---|---|
| Urethane | | | | | | | | |
| 64 mg. × 1 | Croton oil | 24/24 | 100% | 5·0±0·7 | 8 | 24/24 | 100% | 7·0±0·9 |
| 32 mg. × 2 | ,, | 37/37 | 100% | 8·0±0·7 | 6 | 36/37 | 97% | 9·0±0·5 |
| 13 mg. × 5 | ,, | 17/20 | 85% | 2·6±0·6 | 10½ | 13/20 | 65% | 1·7±0·4 |
| 3·2 mg. × 20 | ,, | 21/24 | 87·5% | 2·2±0·4 | 11 | 20/24 | 83% | 3·3±0·8 |

Details of method, as in Table I.

### 3. *Urethane action by oral, subcutaneous, and interperitoneal routes*

The maximum tolerated dose *per os* (64 mg.) was found to cause some deaths when given intraperitoneally. The somewhat smaller dose of 50 mg. was, therefore, chosen for both intraperitoneal and subcutaneous injections. In spite of this slight discrepancy in dosage, when compared with that used orally, it seems clear (Table III) that the dose of urethane, both for initiating action on skin and for complete carcinogenic action on the lung, is of the same order of magnitude for the three routes—orally, intraperitoneally, and subcutaneously.

TABLE III.—*Effect of Route of Administration of Urethane, followed by Standard Croton Oil Treatment on Skin, on Tumour Induction*

| Initiating agent (urethane) (single dose) | Promoting agent | Tumours of skin: Mice bearing papillomas/survivors | | Tumours of skin: Average number papillomas per mouse | Tumours of skin: Average latent period (weeks) | Tumours of lungs: Mice bearing adenomas/survivors | | Tumours of lungs: Average number adenomas per mouse |
|---|---|---|---|---|---|---|---|---|
| 64 mg. *per os* | Croton oil | 24/24 | 100% | 5·0±0·7 | 8 | 24/24 | 100% | 7·0±0·9 |
| 50 mg. intraperitoneally | ,, | 28/28 | 100% | 5·7±0·7 | 8 | 26/28 | 93% | 3·0±0·4 |
| 50 mg. subcutaneously | ,, | 22/22 | 100% | 4·3±0·2 | 10 | 22/22 | 100% | 4·8±0·6 |

Details of method, as in Table I, except for route of administration, as indicated above.

### 4. *Comparisons with skin application (collar experiment)*

Strict quantitative comparisons between the action of urethane by the systemic routes and that resulting from direct skin application was impossible, because of the difficulty in determining the proportion of the applied urethane which is absorbed through the skin. There was, nevertheless, one important aspect of the problem that had to be examined, namely, whether the urethane applied to the skin surface did, in fact, penetrate at all, or whether its action depended on the substance being licked off and swallowed, *thereafter acting systemically.*

To test this, a special plastic collar (Fig. 1) was designed, which would prevent the mouse licking the painted area of skin or the paws that scratched that area. [Preliminary tests, using a highly fluorescent hydrocarbon (3 : 4-benzpyrene) painted on the skin in mice with and without the collar, showed conclusively that the procedure was adequate for preventing the applied material finding its way to the system through the mouth. Fluorescence was readily observed in the contents of the stomach, soon after the skin painting, in mice without the collar, but no trace of fluorescence could be detected in those wearing it.]

Two groups of mice were treated identically, receiving 4 twice-weekly paintings of urethane to the skin, and later, repeated applications of croton oil according to the standard procedure, except that one group was made to wear the collar during the urethane treatment and for one week thereafter (by which time, all the urethane on the skin would either have been absorbed, or would have volatilized). Each mouse was kept in a separate cage during that period. The results (Table IV) do not substantiate the idea that urethane only acted by mouth, since both groups developed tumours of the skin and the lungs. However, the number of tumours per animal was higher in the group without the collar, thus indicating that some of the urethane, at least, normally reaches the systemic circulation by the intestinal route, through licking.

TABLE IV.—*Test for Route of Action of Urethane when Applied Locally to the Skin (Collar Experiment)*

| Initiating agent (urethane applied to skin) | Promoting agent | Tumours of skin: Mice bearing papillomas/survivors | | Tumours of skin: Average number papillomas per mouse | Tumours of skin: Average latent period (weeks) | Tumours of lungs: Mice bearing adenomas/survivors | | Tumours of lungs: Average number adenomas per mouse |
|---|---|---|---|---|---|---|---|---|
| With collar | Croton oil | 22/29 | 76% | 1·0±0·3 | 14½ | 14/29 | 48% | 0·6±0·2 |
| Without collar | ,, | 23/28 | 82% | 1·7±0·3 | 10½ | 25/28 | 89% | 2·0±0·3 |

* Urethane, 40 per cent in acetone, applied twice-weekly for two weeks, to the skin.
Interval between last application of urethane and first application of croton oil : 7 days.
Croton oil treatment, and other details of method, as in Table I.

5. *Variations in interval between initiating and commencement of promoting action*

A single dose of 25 mg. of urethane was given by mouth, as initiator, and skin applications of croton oil, twice-weekly for 26 weeks, served as promotor, the interval between the two, in the different groups, ranging from 30 minutes to 56 days. The results (Table V) failed to show any striking difference in response either in skin tumour or lung tumour incidence, or in the number of tumours per animal, except for one case (56 days interval), in which the lung tumours per animal were significantly higher than in any of the other groups.

EXPLANATION OF PLATE

FIG. 1.—Mouse wearing plastic collar to prevent it licking the painted area of skin.

1

Berenblum and Haran-Ghera.

In a second series, 50 mg. of urethane, injected *subcutaneously*, was followed after 3 and 56 days, respectively, by the standard croton oil treatment on the skin. In this series (Table V), the number of skin papillomas per animal was somewhat higher in the case of the shorter than in that of the longer interval.

TABLE V.—*Influence of Length of Interval Between Initiating Action and Commencement of Promoting Action on Tumour Induction*

| | | Tumours of skin | | | Tumours of lungs | |
|---|---|---|---|---|---|---|
| Initiating agent (urethane) | Interval | Mice bearing papillomas/ survivors | Average number papillomas per mouse | Average latent period (weeks) | Mice bearing adenomas/ survivors | Average number adenomas per mouse |
| 25 mg. *per os* | ½ hour | 20/20 100% | 3·1±0·4 | 9 | 17/20 85% | 2·7±0·5 |
| ,, ,, | 3 hours | 18/20 90% | 2·8±0·5 | 7 | 16/20 80% | 2·7±0·5 |
| ,, ,, | 5 ,, | 18/19 95% | 3·0±0·4 | 9 | 15/19 80% | 1·7±0·3 |
| ,, ,, | 1 day | 16/19 84% | 1·5±0·3 | 11 | 13/19 68% | 2·2±0·6 |
| ,, ,, | 2 days | 18/20 90% | 1·5±0·5 | 11 | 12/20 60% | 1·5±0·4 |
| ,, ,, | 3 ,, | 33/40 82·5% | 1·6±0·2 | 11 | 34/40 85% | 2·7±0·3 |
| ,, ,, | 7 ,, | 36/38 94% | 2·4±0·3 | 11 | 28/38 74% | 2·3±0·4 |
| ,, ,, | 14 ,, | 18/20 90% | 2·4±0·4 | 11 | 17/20 85% | 2·4±0·3 |
| ,, ,, | 56 ,, | 34/37 92% | 2·0±0·3 | 9 | 35/37 94% | 4·8±0·6 |
| 50 mg. s.c. | 3 ,, | 22/22 100% | 4·3±0·2 | 10 | 22/22 100% | 4·8±0·6 |
| ,, ,, | 56 ,, | 16/20 80% | 2·3±0·4 | 10 | 18/20 90% | 3·7±0·65 |

Details of method, as in Table I, except for injection by the subcutaneous route in the last two experiments.

6. *Influence of sex*

Since both male and female mice were used in these experiments, some information about the influence of sex on the present method of carcinogenesis (for this particular strain of mice) might be obtained. Separate data for the two sexes are not shown in Tables I–V, since the number of animals per group would have been too small for statistical evaluation. Since a sex difference nevertheless was suspected, on the grounds that the small observed differences were consistently in the same direction, namely, towards a higher incidence for females, the figures in Table V were pooled, and analysed according to sex, providing the following results :

*Average number papillomas per mouse* : 2·7 ± 0·2 for females ; 1·7 ± 0·2 for males ;

*Average number of lung tumours per mouse* : 3·5 ± 0·3 for females ; 1·7 ± 0·2 for males.

These values are based on 96 females and 97 males.

7. *Possible correlation between the induced tumours of the skin and lungs*

In view of the double action of urethane—initiating action on the skin and complete carcinogenic action on the lungs, and since, under the conditions of the present experiments, the tumour yield in either tissue was often below 100 per cent, it was interesting to determine whether there was any correlation between responsiveness to one action and the other. No such correlation could be detected.

### DISCUSSION

The two-stage mechanism of skin carcinogenesis postulates an *initiating phase*, representing an irreversible transformation of some normal into " latent " or " dormant " tumour cells, followed by a promoting phase, responsible for their conversion into a growing tumour. This concept arose from certain experiments in rabbits, in which tar-induced skin papillomas that had regressed, were made to reappear by further treatment with a variety of stimuli (Rous and Kidd, 1941 ; MacKenzie and Rous, 1941), and from subsequent experiments in mice, in which applications of a carcinogen, insufficient to induce tumours, followed by croton oil treatment, resulted in the appearance of papillomas (Berenblum, 1941 ; Mottram, 1944 ; Berenblum and Shubik, 1947 ; etc.). In both types of experiment, the agent used for initiating action was a complete carcinogen, the promoting component being restricted by limiting the period of treatment (usually to a single application, in the case of the mouse experiment).

Initiating action by the application of urethane to the mouse's skin (Graffi, Vlamynch, Hoffmann, and Schulz, 1953 ; Salaman and Roe, 1953 ; Roe and Salaman, 1954 ; Berenblum and Haran, 1955*b*) provided a refinement in the technique, in that this compound is non-carcinogenic for mouse's skin, even when applied twice-weekly for 43 weeks (Berenblum and Haran, 1955*b*), and also for reasons already referred to in the introduction of this paper. The fact that urethane also acted as an initiating agent for the mouse's skin *when given by mouth* (Haran and Berenblum, 1956), provided certain new approaches to the study of the mechanism of skin carcinogenesis, in that many of the side reactions, resulting from *local* application of an initiating agent, were thereby eliminated.

The first stage of such a study must necessarily be concerned with comparisons between the effectiveness of different routes of administration for systemic action, and with the verification as to whether systemic initiating action was analogous to that arising from local action on the skin. These investigations, reported here, were carried out quantitatively, for additional information.

The fact that urethane given orally, intraperitoneally, and subcutaneously, acted more or less equally effectively for initiating action on the skin (Experiment 3), eliminated the possibility that chemical changes in the gut, and absorption through the intestinal mucosa, modified the effect in any way. The idea that the urethane, applied to the skin, might act indirectly, by being first swallowed through licking (Experiment 4)—an attractive possibility, in view of the absence of any evidence of local irritation—was found to be untenable. Thus, whatever the route by which the urethane enters the system (even by absorption through the skin), it is capable of acting both as an initiator for skin carcinogenesis and as a complete carcinogen for the lung.

The fact that the tumour yield in the skin, resulting from a single feeding of urethane followed by repeated local applications of croton oil to the skin, was determined by the dose of urethane, while the average latent period was not (Experiment 1), is in agreement with the earlier results involving local application of polycyclic aromatic hydrocarbon carcinogens for initiating action (Berenblum and Shubik, 1947), and fails to substantiate some recent criticisms regarding the quantitative relationships of the two-stage mechanism of carcinogenesis. These dealt with two aspects of the problem—(*a*) concerning the quantitative correlation between dose of initiator and yield of tumours, and (*b*) concerning the lack of correlation between dose of initiator and average latent period.

In a series of papers by Roe (1956*a*, 1956*b*) and Salaman and Roe (1956*a*, 1956*b*), experiments are described in which prolongation of the croton oil treatment in the standard experiment (with local application of a hydrocarbon carcinogen as initiator) led to a progressive increase in tumour yield, which seemed contrary to the levelling-off of the curves in the original experiments of Berenblum and Shubik. Since both the carcinogen (used as initiator) and the croton oil (used as promotor) may elicit some " background " carcinogenic action, it might be expected that, after very prolonged treatment, additional tumours would appear as a consequence of these complicating factors. The ideal set-up would be to use a " pure " initiator and a " pure " promotor for testing the hypothesis. We do not yet possess a pure promotor (i.e. *completely* lacking in initiating action), though a pure initiator, in the form of urethane, is now available. The results with urethane, described here, are consistent with the original hypothesis that the *number* of tumours is a function of initiating action while the *latent period* is a function of promoting action. Regarding the latter, the latent period (Experiment 1) varied from group to group, without a consitent correlation with the dose of initiator (as claimed by Klein, 1956).

A number of investigators (Graffi, 1953 ; Druckrey, 1954 ; Roe and Salaman, 1954 ; Klein, 1956) obtained a quantitatively cumulative effect when the same amount of initiator is divided into separate doses ; while some (e.g. Saffiotti and Shubik, 1956) found the small, repeated doses actually more effective than the large, single dose. The present results with urethane given orally (Experiment 2) also suggest a cumulative effect, though not quantitatively. No explanation is available to account for this, though the conditions of the experiment are more complicated than might at first appear, since the dividing up of the dose into several small doses also involves the spreading out of the initiating action over a longer period.

### SUMMARY

The previous observation that urethane, administered by mouth, acted as an initiating agent for skin carcinogenesis in the mouse (i.e. rendering the skin responsive to subsequent promoting action by locally applied croton oil, with the development of papillomas) was confirmed. The intraperitoneal and subcutaneous routes were found to be about equally effective.

The yield of skin papillomas, resulting from the initiating action of orally administered urethane, followed by repeated skin applications of croton oil, was determined by the dose of urethane adminstered (tested over a range of 1–64 mg.), while the average latent period was not related to dose. This result is in keeping with the earlier findings of Berenblum and Shubik (1947), using 9 : 10-dimethyl-1 : 2-benzanthracene, applied locally, as initiator.

Varying the interval between the initiating action of urethane (by mouth) and the commencement of croton oil applications—tested over a range of 30 minutes to 56 days—did not significantly affect the tumour yield in the skin.

By means of a special " collar " experiment, which prevented the mice from licking the skin, it was possible to show that locally applied urethane acted through the skin rather than by being swallowed and absorbed through the intestine

(though a slightly higher tumour yield was observed in mice that were allowed to lick the skin).

The results are discussed in relation to other recent work on the " two-stage mechanism " of skin carcinogenesis.

### REFERENCES

BERENBLUM, I.—(1941) *Cancer Res.*, **1**, 807.
*Idem* AND HARAN, NECHAMA.—(1955*a*) *Ibid.*, **15**, 504.—(1955*b*) *Brit. J. Cancer*, **9**, 453.
*Idem* AND SHUBIK, P.—(1947) *Brit. J. Cancer*, **1**, 383.
DRUCKREY, H.—(1954) *Acta Un. int. Cancr.*, **10**, 29.
GRAFFI, A.—(1953) *Abh. dt. Akad. Wiss., Berlin*, **1**, 1 (quoted by Druckrey, H.).
*Idem*, VLAMYNCH, E., HOFFMANN, F. AND SCHULZ, I.—(1953) *Arch. Geschwulstforsch.*, **5**, 110.
HARAN, NECHAMA AND BERENBLUM, I.—(1956) *Brit. J. Cancer*, **10**, 57.
KLEIN, M.—(1956) *Cancer Res.*, **16**, 123.
MACKENZIE, I. AND ROUS, P.—(1941) *J. exp. Med.*, **73**, 391.
MOTTRAM, J. C.—(1944) *J. Path. Bact.*, **56**, 181.
ROE, F. J. C.—(1956*a*) *Brit. J. Cancer*, **10**, 61.—(1956*b*) *Ibid.*, **10**, 72.
*Idem* AND SALAMAN, M. H.—(1954) *Ibid.*, **8**, 666.
ROUS, P. AND KIDD, J. G.—(1941) *J. exp. Med.*, **73**, 365.
SAFFIOTTI, U. AND SHUBIK, P.—(1956) *J. nat. Cancer Inst.*, **16**, 961.
SALAMAN, M. H. AND ROE, F. J. C.—(1953) *Brit. J. Cancer*, **7**, 472.—(1956*a*) *Ibid.*, **10**, 70.—(1956*b*) *Ibid.*, **10**, 79.